# Accuracy of brain-derived neurotrophic factor levels for differentiating between Taiwanese patients with major depressive disorder or schizophrenia and healthy controls

**DOI:** 10.1371/journal.pone.0212373

**Published:** 2019-02-22

**Authors:** Yu-Jie Chiou, Tiao-Lai Huang

**Affiliations:** Department of Psychiatry, Kaohsiung Chang Gung Memorial Hospital and Chang Gung University College of Medicine, Kaohsiung, Taiwan; National Institutes of Health, UNITED STATES

## Abstract

**Objectives:**

Brain-derived neurotrophic factor (BDNF) has been associated with the psychopathology of both major depressive disorder (MDD) and schizophrenia (SZ). However, studies focusing on the accuracy of BDNF levels to differentiate between these patients and healthy controls (HCs) have been rare.

**Methods:**

Over a discrete ten-year period, we investigated serum BDNF levels in patients with MDD or SZ and compared them to HCs.

**Results:**

We found serum BDNF levels in 224 samples with SZ to be lower than those in 390 HCs samples (p = 0.007), but not lower than those in the 273 samples with MDD. Male MDD patients tended to have lower BDNF levels compared to male HCs (p = 0.083). The receiver operating characteristic curve analysis demonstrated that BDNF levels were moderately accurate in differentiating male MDD patients and female patients with SZ from HCs (AUC = 0.652 and 0.623, respectively). The most adequate cut-off points for BDNF level were 5.11 ng/ml (sensitivity = 81.1%, specificity = 48.5%) and 5.88 ng/ml (sensitivity = 74.1%, specificity = 57.4%), respectively.

**Conclusions:**

Our results support that BDNF demonstrated moderate accuracy in distinguishing male patients with MDD and female patients with SZ from HCs. In the future, greater samples would be required to further confirm these results.

## Introduction

Brain-derived neurotrophic factor (BDNF), which was first purified [1] after the nerve growth factor was discovered [2], has been found to contribute to neurogenesis and neuroplasticity. The mature form of BDNF is a 13-kDa polypeptide that originated from the precursor protein, preproBDNF, in the endoplasmic reticulum. ProBDNF (~32 kDa) is transformed into mature BDNF and BDNF pro-peptide (~17 kDa), the N-terminal fragment of proBDNF, after the signal peptide is split. BDNF can be detected throughout the brain, particularly in the cerebral cortex and hippocampus, brain areas crucial for controlling cognition, mood, and emotions [3]. BDNF can also originate from other tissues besides the brain, such as vascular endothelial cells, smooth muscle, and the liver [4].

Duman, Heninger, and Nestler initially proposed the neurotrophic hypothesis in 1997 [5] and assumed that stress would reduce the expression of BDNF and result in the atrophy of stress-vulnerable hippocampal neurons. Clinical imaging research has supported that the flawed function and diminished volume of these neurons may be associated with depression and has revealed a dwindling size of specific brain areas. These discoveries provided the foundation for an innovative molecular and cellular hypothesis of depression. As a result, major depressive disorder (MDD) was depicted as being secondary to deviant neurogenesis in brain areas that govern emotion and memory, with deviant neurogenesis associated with a decreased expression of BDNF. Later, Karege et al. carried out the pioneer study of BDNF levels in peripheral blood in 2002 [6]. Furthermore, Kuhn, a physicist and science historian, previously observed that, at any given time, researchers in a specific field are inclined to hold similar basic hypotheses about their topic of study [7]. This phenomena rapidly occurred for the neurotrophic hypothesis, which, since its initial proposal, was promptly extended to cover schizophrenia (SZ) by Toyooka in 2002 [8].

The study on peripheral BDNF was originally driven by determining the pathophysiology of mood disorders, but BDNF has recently been applied to serve as a potential biomarker for promoting individualized medicine in psychiatry [9]. BDNF was a manifest option, as its concentration in peripheral blood can be more easily measured than in cerebrospinal fluid (CSF); furthermore, its concentration in plasma and serum is eminently associated with BDNF levels in CSF, since BDNF freely passes the blood–brain barrier [10]. Therefore, peripheral BDNF levels have been investigated as a potential biomarker for diagnosing MDD and SZ. Various reports have capitalized on this “window to the brain”, with an overwhelming majority indicating reduced BDNF concentrations in these subjects [11–13]. BDNF was believed to be engaged in the pathophysiology of MDD [14, 15] and SZ [16–18], and BDNF changes in the blood of patients with MDD or SZ have been supported by a number of meta-analyses [19–22]. Nevertheless, meta-analyses can be impeded by heterogeneous patient populations, the heterogeneity of BDNF assays, and a lack of BDNF standard values. Noticeable inconsistencies in results across papers need to be considered when explaining these data. Furthermore, the capacity, efficacy, and relationship of peripheral BDNF concentrations to disease activity have not been completely established. To clarify these arguments, we designed our study to examine, in a “true-to-life” setting, the hypothesis that BDNF serum levels are reduced in patients with MDD or SZ during acute stages and can thus serve as a biomarker to distinguish these patients from healthy controls (HCs).

### Aims of the study

We investigated whether serum brain-derived neurotrophic factor levels demonstrated any discrepancy between patients with MDD or SZ during acute episodes and HCs. We also examined whether serum brain-derived neurotrophic factor levels could be used for differentiating these acute patients from HCs.

## Methods

### Study design and participants

We adopted a naturalistic study using clinical observation within a ten-year period (Dec. 2003 ~ Nov. 2004, Aug. 2005 ~ Nov. 2006, Aug. 2007 ~ Jul. 2012, Aug. 2014 ~ Jun. 2016, and Dec. 2016 ~ Nov. 2017). Both the patients and HCs were enrolled at Chang Gung Memorial Hospital in Kaohsiung (CGMHK), Taiwan. Patients with MDD or SZ in acute phases were evaluated using the Structured Clinical Interview for DSM-IV Axis I Disorders (SCID) [23] by psychiatrists at CGMHK, including Drs. Tiao-Lai Huang, Chin-Chuen Lin, Yi-Yung Hung, and Meng-Chang Tsai. The patients had not taken any medications for at least one week at the time of their assessment. According to the diagnosis, the severity of symptoms was determined using the 17-item Hamilton Depression Rating Scale (17-item HDRS) [24] or the Positive and Negative Syndrome Rating Scale (PANSS) [25].

Medical students and staff at CGMHK were recruited as the HCs and were evaluated by Tiao-Lai Huang using the SCID [23] or the Chinese Health Questionnaire-12 [26] based on the DSM-III-R, DSM-IV, DSM-IV-TR, and DSM-5 in order to exclude past and present major and minor mental illnesses (illegal substance use disorder, alcohol abuse/dependence, personality disorder, schizophrenia, affective disorder, and anxiety disorder). Some data have already been published [12, 13, 27–29], and we progressively added samples, including patients with MDD or SZ and HCs, to this study. All HCs and patients were screened so that individuals with any systemic disease, including lung, liver, kidney, thyroid, and heart diseases, could be excluded. None of them had either acute infections or allergic reactions. They were not alcohol or drug abusive.

We obtained approval from Chang Gung Medical Foundation Institutional Review Board. All subjects enlisted in this study provided their written informed consent for participation.

### Laboratory analysis

We collected participants’ venous blood samples (5 ml) and assessed serum BDNF levels using an ELISA Kit (BDNF Emax Immunoassay System, Promega Co, USA). All venous blood samples were taken after the patient had fasted for at least 8 hours. Absorbencies were identified utilizing a microtiter plate reader (absorbency at 450 nm), and intra-assay and inter-assay variations were both less than 10%.

### Statistical analysis

Subjects were divided into different diagnostic groups (i.e., patients with MDD, patients with SZ, or HCs). We applied student’s t-tests to evaluate such parameters as age, sex, and body mass index (BMI) of both the patients and the HCs. The relationship between BDNF levels and severity scores was examined through the Pearson’s correlation test. Previous studies have indicated that parameters like age and sex can influence circulating BDNF levels in peripheral blood [30]. BDNF levels of the patient and control groups were then compared using an analysis of covariance (ANCOVA) with sex, age, and BMI adjustments for group mean differences in the different groups and sexes.

We investigated the estimation validity of BDNF to discriminate patients from HCs by using receiver operating characteristic (ROC) analyses with calculations of the area under the ROC curve (AUC), sensitivity, and specificity. A suitable BDNF cutoff level was defined as the level that demonstrated the greatest sensitivity among the greatest values on the Youden index [(sensitivity + specificity) - 1]. The Youden index is considered an efficient index for determining a suitable cutoff score [31].

All results are shown as mean ± standard deviation. We calculated data analysis using IBM SPSS Statistics 12. Two-tailed significance values were adopted, and -significance levels were determined at a value of 0.05.

## Results

### Demographic data

Overall, we recruited 497 patient samples and 390 HC samples. The patient samples consisted of 273 major depressive disorder and 224 schizophrenia diagnoses.

Table 1 depicts the significant differences in age and sex between depressed patients and HCs (39.4 ± 11.8 years vs. 31.3 ± 6.7 years, df = 393.7, p < .001; 0.24 ± 0.43 vs. 0.37 ± 0.48, df = 624.6, p < .001), as well as the significant age differences between the male subgroups (41.1 ± 10.2 years vs. 30.9 ± 5.9 years; df = 85.6, p < .001) and female subgroups (38.9 ± 12.2 years vs. 31.5 ± 7.1 years; df = 317.1, p < .001). Only the female subgroups demonstrated a significant difference in BMI (22.3 ± 4.2 kg/m^2^ vs. 21.3 ± 3.0 kg/m^2^; df = 366.7, p = 0.006). The average 17-item HDRS score was 31.9 ± 5.6.

**Table 1:** Serum BDNF levels and demographic data of patients with major depressive disorder and healthy controls.

Diagnostic groups		Age (years)	Sex^♠^	BMI (kg/m^2^)	Serum BDNF levels (ng/ml)	17-item HDRS
**T**	**PATIENTS** *(n = 273)*	39.4 ± 11.8	0.24 ± 0.43	22.7 ± 4.3	8.3 ± 6.0	31.9 ± 5.6
	**Healthy controls** *(n = 390)*	31.3 ± 6.7	0.37 ± 0.48	22.4 ± 3.6	9.5 ± 6.7	N.A.
**P-value**		< .001*	< .001*	0.441	0.145	
**M**	**PATIENTS** *(n = 66)*	41.1 ± 10.2	N.A.	23.9 ± 4.2	7.2 ± 6.5	32.7 ± 4.9
	**Healthy controls** *(n = 143)*	30.9 ± 5.9		24.4 ± 3.7	9.2 ± 5.4	N.A.
**P-value**		< .001*		0.405	0.083	
**F**	**PATIENTS** *(n = 207)*	38.9 ± 12.2		22.3 ± 4.2	8.7 ± 5.7	31.6 ± 5.8
	**Healthy controls** *(n = 247)*	31.5 ± 7.1		21.3 ± 3.0	9.7 ± 7.4	N.A.
**P-value**		< .001*		0.006*	0.368	

T = Total. M = Male. F = Female. PATIENTS = Patients with major depressive disorder. BMI = body mass index. BDNF = brain-derived neurotrophic factor. 17-item HDRS, 17-item Hamilton Depression Rating Scale.

^♠^ Female = 0. Male = 1

*P < 0.05

Table 2 shows that the schizophrenia patients were significantly older and had a greater proportion of male individuals and higher BMI than HCs (33.8 ± 10.1 years vs. 31.3 ± 6.7 years, df = 335.6, p = 0.001; 0.52 ± 0.50 vs. 0.37 ± 0.48, df = 450.7, p < .001; 23.8 ± 4.4 kg/m^2^ vs. 22.4 ± 3.6 kg/m^2^, df = 389.9, p < .001). Only the female subgroups demonstrated significant differences in age and BMI (35.2 ± 11.2 years vs. 31.5 ± 7.1 years, df = 145.5, p = 0.002; 22.8 ± 4.2 kg/m^2^ vs. 21.3 ± 3.0 kg/m^2^, df = 158.5, p = 0.001). The average PANSS score was 121.8 ± 20.0.

**Table 2:** Serum BDNF levels and demographic data of patients with schizophrenia and healthy controls.

Diagnostic groups		Age (years)	Sex^♠^	BMI (kg/m^2^)	Serum BDNF levels (ng/ml)	PANSS total score
**T**	**PATIENTS** *(n = 224)*	33.8 ± 10.1	0.52 ± 0.50	23.8 ± 4.4	8.0 ± 5.6	121.8 ± 20.0
	**Healthy controls** *(n = 390)*	31.3 ± 6.7	0.37 ± 0.48	22.4 ± 3.6	9.5 ± 6.7	N.A.
**P-value**		0.001*	< .001*	< .001*	0.007*	
**M**	**PATIENTS** *(n = 116)*	32.4 ± 8.8	N.A.	24.7 ± 4.5	8.4 ± 5.5	120.3 ± 20.4
	**Healthy controls** *(n = 143)*	30.9 ± 5.9		24.4 ± 3.7	9.2 ± 5.4	N.A.
**P-value**		0.127		0.541	0.269	
**F**	**PATIENTS** *(n = 108)*	35.2 ± 11.2		22.8 ± 4.2	7.5 ± 5.7	123.3 ± 19.6
	**Healthy controls** *(n = 247)*	31.5 ± 7.1		21.3 ± 3.0	9.7 ± 7.4	N.A.
**P-value**		0.002*		0.001*	0.008*	

T = Total. M = Male. F = Female. PATIENTS = Patients with schizophrenia. BMI = body mass index. BDNF = brain-derived neurotrophic factor. PANSS = Positive and Negative Syndrome Scale.

^♠^ Female = 0. Male = 1

*P < 0.05

### BDNF levels in patients and HCs

The ANCOVAs were established with diagnostic groups as the independent variables and BDNF levels as the dependent variables; age, sex and BMI were the covariates.

Table 1 indicates that no significant difference was observed between depressed patients and HCs (8.3 ± 6.0 ng/ml vs. 9.5 ± 6.7 ng/ml, F = 2.132, p = 0.145). Depressed men tended to have lower BDNF levels (7.2 ± 6.5 ng/ml vs. 9.2 ± 5.4 ng/ml, F = 3.044, p = 0.083), but not depressed women (8.7 ± 5.7 ng/ml vs. 9.7 ± 7.4 ng/ml, F = 0.813, p = 0.368). Pearson’s correlation test revealed that 17-item HDRS scores had no correlation with BDNF levels (γ = 0.015, P = 0.802).

Table 2 reveals that schizophrenic patients had significantly lower BDNF levels than HCs (8.0 ± 5.6 ng/ml vs. 9.5 ± 6.7 ng/ml, F = 7.414, p = 0.007). Schizophrenic women had significantly lower BDNF levels (7.5 ± 5.7 ng/ml vs. 9.7 ± 7.4 ng/ml, F = 7.163, p = 0.008), but schizophrenic men did not (8.4 ± 5.5 ng/ml vs. 9.2 ± 5.4 ng/ml, F = 1.225, p = 0.269). Pearson’s correlation test indicated that PANSS scores were not related to BDNF levels (γ = 0.025, P = 0.714).

### BDNF levels in differentiating between patients and HCs

**Fig 1:**
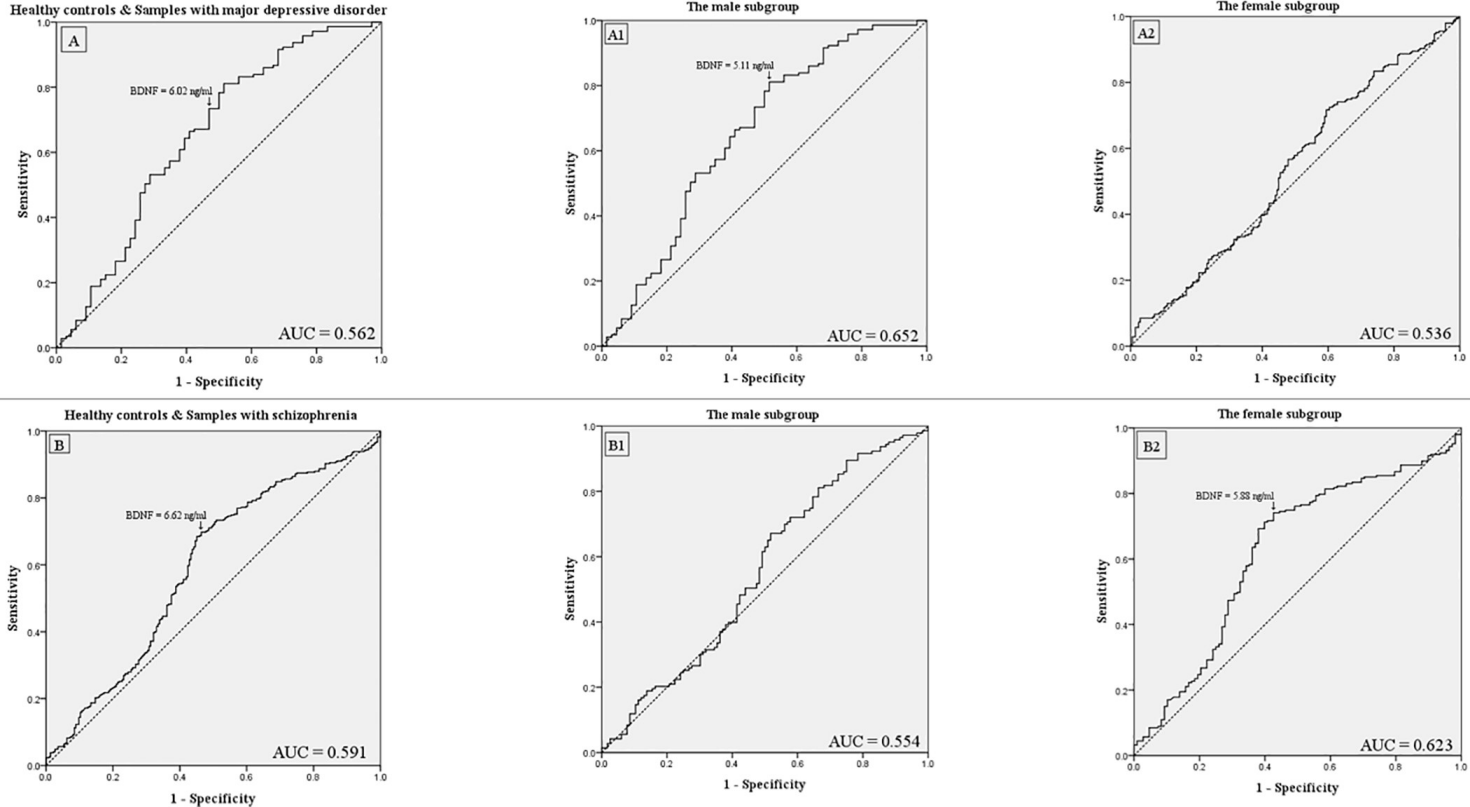
Receiver operating characteristic (ROC) curve. Receiver operating characteristic (ROC) curve using brain-derived neurotrophic factor (BDNF) to discriminate healthy controls (N = 390) from: (A) samples with major depressive disorder (N = 273) and (B) samples with schizophrenia (N = 224). The subgroups A1 and B1 represent the male subgroups, and the subgroups A2 and B2 represent the female subgroups.

The ROC curve analysis (Fig 1A) shows that BDNF levels had a poor differential efficacy for depressed patients and HCs (AUC = 0.562, standard error = 0.023, asymptotic 95% confidential interval = 0.516 to 0.607). The optimal cut-off point for BDNF level was 6.02 ng/ml (sensitivity = 72.8%, specificity = 41.8%). BDNF levels demonstrated moderate diagnostic power in the male subgroup (AUC = 0.652, sensitivity = 81.1% and specificity = 48.5% at the BDNF level of 5.11 ng/ml) (Fig 1A1), but not in the female subgroup (AUC = 0.536) (Fig 1A2).

BDNF levels (Fig 1B) features acceptable diagnostic power for distinguishing between schizophrenic patients and HCs (AUC = 0.591, standard error = 0.024, asymptotic 95% confidential interval = 0.543 to 0.639) The most apt cut-off point for BDNF level was 6.62 ng/ml (sensitivity = 68.5%, specificity = 54.9%). BDNF levels have an acceptable differential efficacy in the female subgroup (AUC = 0.623, sensitivity = 74.1%, specificity = 57.4% at the BDNF level of 5.88 ng/ml) (Fig 1B2), but not in the male subgroup (AUC = 0.554) (Fig 1B1).

## Discussion

This study, which concentrated on patients in acute states, resulted in the following major findings: (1) Patients with SZ had lower serum BDNF levels than HCs. (2) Male MDD patients tended to have lower BDNF levels than male HCs. (3) The receiver operating characteristic curve analysis showed that BDNF levels demonstrated moderate accuracy for distinguishing male patients with MDD and female patients with SZ from HCs.

### BDNF levels in patients with MDD or SZ and HCs

We demonstrated that the serum BDNF levels in patients with SZ were significantly lower than those in HCs; this finding agreed with the outcome of another recent meta-analysis [19]. Like results previously reported [13], our results reinforce the hypothesis that down-regulated BDNF expression not only plays a pivotal role in neuroplasticity, developmental processes, and reconstruction, but is also associated with the pathophysiology of SZ.

In contrast, serum BDNF levels in MDD patients indicated no significant difference compared to HCs, and we observed a trend towards lower BDNF levels in male MDD patients compared to male HCs, which did not agree with our previous reports [12, 28] and other recent meta-analyses [19, 20, 22]. In their study, Molendijk et al. [20] found that year of publication and sample size could forecast between-study heterogeneity, with more recently published results and bigger samples being associated with lower between-group differences. Reports with positive results are more likely to be published than reports with negative results, a publication bias that was evident in funnel plots [20]. Furthermore, the heterogeneity may have arisen from between-sample features that were not examined in most studies, such as sleep problems [32], smoking [33], or seasonality [34]. As depression is a heterogeneous psychiatric disorder [35], heterogeneity in results may also be caused by various clinical features of the samples. Bus et al. also indicated that persistently depressed and remitted patients had significantly reduced BDNF levels over time, but the BDNF reduction in patients with incident depression resembled BDNF levels in HCs [36]. Furthermore, Cain et al. [37] recently studied the individual circadian rhythm of plasma BDNF and found that plasma BDNF can alter significantly in just one day in both men and women, with top timing that extraordinarily varied among subjects and the time of acrophase being independent of the clock time. While the aforementioned data need to be replicated in separate studies with larger sample sizes, we believe that BDNF can fill an essential role in leading to the dynamic neurobiological and clinical aberration found in MDD and SZ.

We found 17-item HDRS and PANSS scores not to be related to BDNF levels among patients with MDD or SZ, which was in line with some previous studies [12, 13, 21], but differed with others [10, 38–43]. However, these cross-sectional research projects only focused on the relationship between the severity of clinical symptoms and BDNF levels at the onset of disease and thus were unable to determine causal association.

Although the aforementioned results need to be repeated in future studies with larger samples, we believe that BDNF plays a pivotal role in eliciting the dynamic clinical and neurobiological changes observed in various stages of illness.

### The accuracy of BDNF in distinguishing patients with MDD or SZ from HCs

An exact diagnosis of mental illness is critical for providing proper treatment. However, an appropriate biomarker has yet to be found that can be applied to diagnose any psychiatric disorder, and diagnostic criteria still primarily relies on clinical interviews. Furthermore, the present diagnostic criteria has restrictions, as psychiatrists diagnosing mental illness solely on the basis of interviews can be biased. Treatment may also be improper due to inaccurate diagnosis. Previous scholars recently proposed that BDNF could be a physiopathological biomarker in psychiatry [19], but research examining the role of BDNF in differentiating patients with MDD or SZ from HCs has been scarce. We demonstrated that BDNF levels had moderate accuracy in distinguishing male patients with MDD and female patients with SZ from HCs. Nevertheless, BDNF levels had no validity in distinguishing female patients with MDD and male patients with SZ from HCs. The discrepancy between women and men may be influenced by sex hormones [28, 30, 44]. BDNF levels could not ideally differentiate patients with MDD or SZ from HCs and had a poor diagnostic power of AUC under 0.6. Chen et al. [45] also failed to exceptionally distinguish these patients from HCs solely using BDNF levels. The specificity of the BDNF was not satisfied, the reason may be the relative sample size. Furthermore, some studies demonstrated that the model of combination of BDNF serum level and RNA expression can distinguish the psychiatric state [46]. Thus, only BDNF serum level might be difficult to distinguish the groups [46]. Multiple-assay methods may play a better role as biomarker, as has recently been investigated [45, 47].

This study has some limitations that should be mentioned at this point. The primary limitation of this study is that BDNF levels were detected in serum and thus displayed an indirect measurement of brain BDNF levels. The genuine level of brain BDNF expression and its effect on MDD or SZ requires further research to draw firmer conclusions. Another limitation is that the impact potentially caused by sleep problems [32], smoking [33] or seasonality [34], all of which have been reported to affect BDNF expression, were not studied. Third, some epidemiological differences were observed between patients and HCs. However, these variables were addressed through ANCOVA. Fourth, the ELISA kit utilized in this study recognized both mature form of BDNF and precursor of BDNF due to the limited specificity of its BDNF antibody. In the future studies, we will use Elisa kit that only detect mature form of BDNF. Lastly, one week is insufficient to preclude the effects of medication on BDNF level.

Our results demonstrated that patients with SZ had lower serum BDNF levels than HCs, and male patients tended to have lower BDNF levels than male HCs. BDNF levels demonstrated moderate diagnostic power in the male subgroup, but not in the female subgroup. BDNF levels have an acceptable differential efficacy in the female subgroup, but not in the male subgroup. In the future, studies with larger samples will be required to study the ability of BDNF to differentiate between patients with MDD or SZ and HCs.

## Supporting information

S1 File De-identified to your manuscript. (XLSX)

## References

[1] Barde YA, Edgar D, Thoenen H. Purification of a new neurotrophic factor from mammalian brain. EMBO J. 1982;1(5):549–53. 7188352 10.1002/j.1460-2075.1982.tb01207.x PMC553086

[2] Levi-Montalcini R, Hamburger V. Selective growth stimulating effects of mouse sarcoma on the sensory and sympathetic nervous system of the chick embryo. J Exp Zool. 1951;116(2):321–61. . 14824426 10.1002/jez.1401160206

[3] Post RM. Role of BDNF in bipolar and unipolar disorder: clinical and theoretical implications. J Psychiatr Res. 2007;41(12):979–90. 10.1016/j.jpsychires.2006.09.009 . 17239400

[4] Cassiman D, Denef C, Desmet VJ, Roskams T. Human and rat hepatic stellate cells express neurotrophins and neurotrophin receptors. Hepatology. 2001;33(1):148–58. 10.1053/jhep.2001.20793 . 11124831

[5] Duman RS, Heninger GR, Nestler EJ. A molecular and cellular theory of depression. Arch Gen Psychiatry. 1997;54(7):597–606. . 9236543 10.1001/archpsyc.1997.01830190015002

[6] Karege F, Perret G, Bondolfi G, Schwald M, Bertschy G, Aubry JM. Decreased serum brain-derived neurotrophic factor levels in major depressed patients. Psychiatry Res. 2002;109(2):143–8. . 11927139 10.1016/s0165-1781(02)00005-7

[7] Kuhn TS. Historical structure of scientific discovery. Science. 1962;136(3518):760–4. . 14460344 10.1126/science.136.3518.760

[8] Toyooka K, Asama K, Watanabe Y, Muratake T, Takahashi M, Someya T, et al Decreased levels of brain-derived neurotrophic factor in serum of chronic schizophrenic patients. Psychiatry Res. 2002;110(3):249–57. . 12127475 10.1016/s0165-1781(02)00127-0

[9] Fernandes BS, Gama CS, Kauer-Sant'Anna M, Lobato MI, Belmonte-de-Abreu P, Kapczinski F. Serum brain-derived neurotrophic factor in bipolar and unipolar depression: a potential adjunctive tool for differential diagnosis. J Psychiatr Res. 2009;43(15):1200–4. 10.1016/j.jpsychires.2009.04.010 . 19501841

[10] Pillai A, Kale A, Joshi S, Naphade N, Raju MS, Nasrallah H, et al Decreased BDNF levels in CSF of drug-naive first-episode psychotic subjects: correlation with plasma BDNF and psychopathology. Int J Neuropsychopharmacol. 2010;13(4):535–9. 10.1017/S1461145709991015 . 19941699

[11] Nuernberg GL, Aguiar B, Bristot G, Fleck MP, Rocha NS. Brain-derived neurotrophic factor increase during treatment in severe mental illness inpatients. Transl Psychiatry. 2016;6(12):e985 10.1038/tp.2016.227 27959329 PMC5290335

[12] Chiou YJ, Huang TL. Serum Brain-Derived Neurotrophic Factors in Taiwanese Patients with Drug-Naive First-Episode Major Depressive Disorder: Effects of Antidepressants. Int J Neuropsychopharmacol. 2017;20(3):213–8. 10.1093/ijnp/pyw096 27811136 PMC5408974

[13] Chiou YJ, Huang TL. Serum brain-derived neurotrophic factors in Taiwanese patients with drug-naive first-episode schizophrenia: Effects of antipsychotics. World J Biol Psychiatry. 2017;18(5):382–91. 10.1080/15622975.2016.1224925 . 27643618

[14] Youssef MM, Underwood MD, Huang YY, Hsiung SC, Liu Y, Simpson NR, et al Association of BDNF Val66Met Polymorphism and Brain BDNF Levels with Major Depression and Suicide. Int J Neuropsychopharmacol. 2018;21(6):528–38. 10.1093/ijnp/pyy008 . 29432620 PMC6007393

[15] Huang TL, Lin CC. Advances in biomarkers of major depressive disorder. Adv Clin Chem. 2015;68:177–204. 10.1016/bs.acc.2014.11.003 . 25858873

[16] Man L, Lv X, Du XD, Yin G, Zhu X, Zhang Y, et al Cognitive impairments and low BDNF serum levels in first-episode drug-naive patients with schizophrenia. Psychiatry Res. 2018;263:1–6. 10.1016/j.psychres.2018.02.034 . 29482040

[17] Zhang Y, Fang X, Fan W, Tang W, Cai J, Song L, et al Brain-derived neurotrophic factor as a biomarker for cognitive recovery in acute schizophrenia: 12-week results from a prospective longitudinal study. Psychopharmacology (Berl). 2018;235(4):1191–8. 10.1007/s00213-018-4835-6 . 29392373

[18] Heitz U, Papmeyer M, Studerus E, Egloff L, Ittig S, Andreou C, et al Plasma and serum brain-derived neurotrophic factor (BDNF) levels and their association with neurocognition in at-risk mental state, first episode psychosis and chronic schizophrenia patients. World J Biol Psychiatry. 2018:1–10. 10.1080/15622975.2018.1462532 . 29938562

[19] Fernandes BS, Berk M, Turck CW, Steiner J, Goncalves CA. Decreased peripheral brain-derived neurotrophic factor levels are a biomarker of disease activity in major psychiatric disorders: a comparative meta-analysis. Mol Psychiatry. 2014;19(7):750–1. 10.1038/mp.2013.172 . 24342989

[20] Molendijk ML, Spinhoven P, Polak M, Bus BA, Penninx BW, Elzinga BM. Serum BDNF concentrations as peripheral manifestations of depression: evidence from a systematic review and meta-analyses on 179 associations (N = 9484). Mol Psychiatry. 2014;19(7):791–800. 10.1038/mp.2013.105 . 23958957

[21] Fernandes BS, Steiner J, Berk M, Molendijk ML, Gonzalez-Pinto A, Turck CW, et al Peripheral brain-derived neurotrophic factor in schizophrenia and the role of antipsychotics: meta-analysis and implications. Mol Psychiatry. 2015;20(9):1108–19. 10.1038/mp.2014.117 . 25266124

[22] Kishi T, Yoshimura R, Ikuta T, Iwata N. Brain-Derived Neurotrophic Factor and Major Depressive Disorder: Evidence from Meta-Analyses. Front Psychiatry. 2017;8:308 10.3389/fpsyt.2017.00308 29387021 PMC5776079

[23] First MB, Spitzer RL, Gibbon M, Williams JBW. User's Guide for the Structured Clinical Interview for DSM-IV Axis I Disorders-Clinician Version (SCID-CV). Washington, DC: American Psychiatric Press 1997.

[24] Hamilton M. A rating scale for depression. J Neurol Neurosurg Psychiatry. 1960;23:56–62. 14399272 10.1136/jnnp.23.1.56 PMC495331

[25] Kay SR, Opler LA, Lindenmayer JP. The Positive and Negative Syndrome Scale (PANSS): rationale and standardisation. Br J Psychiatry Suppl. 1989;(7):59–67. . 2619982

[26] Chong MY, Wilkinson G. Validation of 30- and 12-item versions of the Chinese Health Questionnaire (CHQ) in patients admitted for general health screening. Psychol Med. 1989;19(2):495–505. . 2788292 10.1017/s0033291700012526

[27] Huang TL, Lee CT. Associations between serum brain-derived neurotrophic factor levels and clinical phenotypes in schizophrenia patients. J Psychiatr Res. 2006;40(7):664–8. 10.1016/j.jpsychires.2005.11.004 . 16386272

[28] Huang TL, Lee CT, Liu YL. Serum brain-derived neurotrophic factor levels in patients with major depression: effects of antidepressants. J Psychiatr Res. 2008;42(7):521–5. 10.1016/j.jpsychires.2007.05.007 . 17585940

[29] Chen CC, Huang TL. Effects of antipsychotics on the serum BDNF levels in schizophrenia. Psychiatry Res. 2011;189(3):327–30. 10.1016/j.psychres.2011.01.011 . 21320726

[30] Elfving B, Buttenschon HN, Foldager L, Poulsen PH, Andersen JH, Grynderup MB, et al Depression, the Val66Met polymorphism, age, and gender influence the serum BDNF level. J Psychiatr Res. 2012;46(9):1118–25. 10.1016/j.jpsychires.2012.05.003 . 22682508

[31] Perkins NJ, Schisterman EF. The inconsistency of "optimal" cutpoints obtained using two criteria based on the receiver operating characteristic curve. Am J Epidemiol. 2006;163(7):670–5. 10.1093/aje/kwj063 16410346 PMC1444894

[32] Giese M, Unternahrer E, Huttig H, Beck J, Brand S, Calabrese P, et al BDNF: an indicator of insomnia? Mol Psychiatry. 2014;19(2):151–2. 10.1038/mp.2013.10 23399916 PMC3903111

[33] Bus BA, Molendijk ML, Penninx BJ, Buitelaar JK, Kenis G, Prickaerts J, et al Determinants of serum brain-derived neurotrophic factor. Psychoneuroendocrinology. 2011;36(2):228–39. 10.1016/j.psyneuen.2010.07.013 . 20702043

[34] Molendijk ML, Haffmans JP, Bus BA, Spinhoven P, Penninx BW, Prickaerts J, et al Serum BDNF concentrations show strong seasonal variation and correlations with the amount of ambient sunlight. PLoS One. 2012;7(11):e48046 10.1371/journal.pone.0048046 23133609 PMC3487856

[35] Rush AJ. The varied clinical presentations of major depressive disorder. J Clin Psychiatry. 2007;68 Suppl 8:4–10. . 17640152

[36] Bus BA, Molendijk ML, Tendolkar I, Penninx BW, Prickaerts J, Elzinga BM, et al Chronic depression is associated with a pronounced decrease in serum brain-derived neurotrophic factor over time. Mol Psychiatry. 2015;20(5):602–8. 10.1038/mp.2014.83 . 25155878

[37] Cain SW, Chang AM, Vlasac I, Tare A, Anderson C, Czeisler CA, et al Circadian Rhythms in Plasma Brain-derived Neurotrophic Factor Differ in Men and Women. J Biol Rhythms. 2017;32(1):75–82. 10.1177/0748730417693124 . 28326910

[38] Buckley PF, Pillai A, Evans D, Stirewalt E, Mahadik S. Brain derived neurotropic factor in first-episode psychosis. Schizophr Res. 2007;91(1–3):1–5. 10.1016/j.schres.2006.12.026 17306505 PMC1933504

[39] Rizos EN, Rontos I, Laskos E, Arsenis G, Michalopoulou PG, Vasilopoulos D, et al Investigation of serum BDNF levels in drug-naive patients with schizophrenia. Prog Neuropsychopharmacol Biol Psychiatry. 2008;32(5):1308–11. 10.1016/j.pnpbp.2008.04.007 . 18502013

[40] Chen DC, Wang J, Wang B, Yang SC, Zhang CX, Zheng YL, et al Decreased levels of serum brain-derived neurotrophic factor in drug-naive first-episode schizophrenia: relationship to clinical phenotypes. Psychopharmacology (Berl). 2009;207(3):375–80. 10.1007/s00213-009-1665-6 . 19787338

[41] Xiu MH, Hui L, Dang YF, Hou TD, Zhang CX, Zheng YL, et al Decreased serum BDNF levels in chronic institutionalized schizophrenia on long-term treatment with typical and atypical antipsychotics. Prog Neuropsychopharmacol Biol Psychiatry. 2009;33(8):1508–12. 10.1016/j.pnpbp.2009.08.011 . 19720106

[42] Lee AH, Lange C, Ricken R, Hellweg R, Lang UE. Reduced brain-derived neurotrophic factor serum concentrations in acute schizophrenic patients increase during antipsychotic treatment. J Clin Psychopharmacol. 2011;31(3):334–6. 10.1097/JCP.0b013e31821895c1 . 21508862

[43] Sotiropoulou M, Mantas C, Bozidis P, Marselos M, Mavreas V, Hyphantis T, et al BDNF serum concentrations in first psychotic episode drug-naive schizophrenic patients: associations with personality and BDNF Val66Met polymorphism. Life Sci. 2013;92(4–5):305–10. 10.1016/j.lfs.2013.01.008 . 23333821

[44] Halbreich U, O'Brien PM, Eriksson E, Backstrom T, Yonkers KA, Freeman EW. Are there differential symptom profiles that improve in response to different pharmacological treatments of premenstrual syndrome/premenstrual dysphoric disorder? CNS Drugs. 2006;20(7):523–47. 10.2165/00023210-200620070-00001 . 16800714

[45] Chen S, Jiang H, Liu Y, Hou Z, Yue Y, Zhang Y, et al Combined serum levels of multiple proteins in tPA-BDNF pathway may aid the diagnosis of five mental disorders. Sci Rep. 2017;7(1):6871 10.1038/s41598-017-06832-6 28761093 PMC5537244

[46] Li Z, Zhang C, Fan J, Yuan C, Huang J, Chen J, et al Brain-derived neurotrophic factor levels and bipolar disorder in patients in their first depressive episode: 3-year prospective longitudinal study. Br J Psychiatry. 2014;205(1):29–35. 10.1192/bjp.bp.113.134064 . 24764546

[47] Li C, Tao H, Yang X, Zhang X, Liu Y, Tang Y, et al Assessment of a combination of Serum Proteins as potential biomarkers to clinically predict Schizophrenia. Int J Med Sci. 2018;15(9):900–6. 10.7150/ijms.24346 30008602 PMC6036096

